# How to come to terms with facing death: a qualitative study examining the experiences of patients with terminal Cancer

**DOI:** 10.1186/s12904-019-0417-6

**Published:** 2019-04-04

**Authors:** Ayumi Kyota, Kiyoko Kanda

**Affiliations:** Department of Nursing, Gunma University Graduate School of Health Sciences, 3-39-22 Showa-machi, Maebashi, Gunma 371-8514 Japan

**Keywords:** Palliative home care, Palliative care units, Terminal cancer patients, Mental stabilization, Life and death, Qualitative study

## Abstract

**Background:**

Cancer patients who have reached the terminal stage despite attempts at treatment are likely to experience various problems, particularly as they encounter increasing difficulty in doing what they were able to do easily, and their physical symptoms increase as the disease advances. The purpose of this study is to explore how terminal cancer patients who have not clearly expressed a depressed mood or intense grief manage their feelings associated with anxiety and depression.

**Methods:**

Eleven terminally ill patients with cancer who were receiving symptom-relieving treatment at home or in palliative care units were interviewed. Interviews were generally conducted weekly, two to five times for each participant. In total, 33 interviews were conducted, and the overall interview time was 2027 min. Data were analyzed via qualitative methods.

**Results:**

The following five themes were extracted regarding the experience of managing feelings associated with anxiety and depression when facing death: “I have to accept that I have developed cancer,” “I have to accept the undeniable approach of my own death,” “I have to accept my need for assistance,” “I have to accept this unsatisfactory circumstance” and “I have to accept this as my destiny and an outcome of my life.”

**Conclusion:**

The present study revealed key themes related to how patients come to terms with their impending death. Nurses are required to comprehend the patients’ complicated mental patterns that are expressed in their daily languages. Furthermore, the findings clarify the necessity for nurses to help patients understand the acceptance of a terminal disease state during a patient’s final days.

## Background

In 2011, 37.4% patients who died worldwide could have benefitted from specialist palliative care [1]. Additionally, more than 34% of adults in need of palliative care eventually died due to cancer [1]. Thus, the need for palliative care for terminal cancer patients is high worldwide.

In 1967, for the first time in the world, Cicely Saunders founded the modern form of hospice, based on her view that it is important for terminally ill patients to be cared for by experts [2]. In the 1970s, thanatology and death studies spread widely, primarily in Europe and the USA.

There were 357 palliative care units (PCUs) and 7184 beds to serve Japan’s population of 127 million [3]. More than 80% of cancer deaths occur in general wards; home hospice and PCUs accounted for less than 20% of cancer deaths [4]. PCUs have beds for cancer and acquired immune-deficiency syndrome patients with the average length of stay of 33 days, and the average deceased patient discharge rate is 84% [3]. Meanwhile, only 991 hospitals (or 13.3%) have palliative care teams for hospitalized patients and outpatients with cancer [5]. Patients who can receive specialist palliative care, such as PCUs and palliative care teams, are limited to specific hospitals and few home hospice clinics. To address this issue, the Japanese government adopted the policy of promoting palliative care and home hospice [6]. The number of cancer patients who will spend their final days at home or in PCUs is expected to increase, particularly in high-income countries [7, 8]; this shift is due to advancements in high-quality palliative care provided as a result of the trends of increasing patient needs and demand for reductions in healthcare expenditures [7, 8].

Professionals providing palliative care to terminally ill patients have focused on what patients feel, think, and desire regarding their impending death [9]. The following theories have been proposed regarding the way in which terminally ill patients accept death. Acceptance is an active process where the patient becomes open to and acknowledges all aspects of his or her current situation, whether physical or emotional, to make the most of the time he or she has left [10]. For instance, Buckman [11] proposed a three-stage theory that included fear, a sense of sin, desire and despair, and humour, which are often seen in near-death situations. The Patient Health Engagement model not only describes the patient’s emotional adjustment to the disease and diagnosis, but also tries to describe how the self-concept is reconfigured [12]. Acceptance-based interventions, from approaches such as Acceptance and Commitment Therapy, are also being applied to patients in palliative care [13]. However, terminally ill patients do not always accept death, and many require mental healthcare in order to manage the negative emotions generated by the awareness of their imminent mortality.

Healthcare professionals are keenly interested in assisting with the anxiety and spiritual suffering experienced by terminal cancer patients [14, 15]. The trajectories of functional decline at the end of life are substantially different between patients diagnosed with cancer and other advanced organ diseases [16]. Cancer patients who have reached a terminal stage despite treatment attempts suffer in various ways; physical pain, loss of meaning, loss of autonomy, feeling of being a burden, fear of future suffering, and worry occur frequently among patients with terminal cancer [17]. Healthcare professionals, including nurses, have a responsibility to understand this suffering and its impact on their patients’ lives. Nurses have the important task of screening patients for specialized mental healthcare that may be necessary. Therefore, some patients are referred to mental healthcare professionals, such as grief and bereavement counsellors, social workers, psychologists, and psychiatrists.

However, not all patients with terminal cancer are referred for specialized mental healthcare; approximately 24% of terminal cancer patients have been diagnosed with an anxiety or depressive disorder [18]. This value is higher than the healthy people population [19]. The desire for death in terminally cancer patients is associated with depression and anxiety; however, patients with no mental disorders may also desire death [18]. Patients who do not receive professional mental healthcare must manage various emotions such as sorrow, anxiety, and depression, with the help of their daily care nurses. Several transdiagnostic psychological treatments for anxiety and depressive disorders have been reported [20]. However, there are not well established methods for nursing support to manage the feelings of anxiety and depression associated with terminal cancer patients who have not clearly expressed a depressed mood or intense grief.

It is necessary for nurses in the outpatient section and wards to assess patients’ mental health and consult with doctors in order to request relevant care, but it is not easy. Symptom assessment scales [21, 22] are often used by nurses to identify the need for special interventions such as by palliative care teams. However, it has also been pointed out that there is a lack of consensus in the cut-offs of symptom assessment tools and timing for outpatient palliative care referral [23]. Therefore, it is necessary to assess the mental state of patients not only on a scale but also based on the patients’ own narration.

The purpose of this study is to explore how terminal cancer patients who have not clearly expressed a depressed mood or intense grief manage their feelings associated with anxiety and depression. This research will deepen nurses’ understanding and clarify how the feelings of patients with no obvious problems can be managed. This study is expected to lead to the provision of appropriate nursing assistance and advances in palliative nursing skills.

## Methods

### Study design

The present study was based on a psychophenomenological approach, which is a descriptive phenomenological approach originating from Husserl’s philosophy. The descriptive phenomenology is used to illuminate poorly understood aspects of experiences; it aims to identify and describe the meanings behind people’s experiences [24, 25]. Phenomenology is a highly appropriate and suitable methodology for mental health research, since it includes people’s experiences and enables silent voices to be heard [26]. The psychophenomenological approach is designed to gain a deep understanding regarding the lived experiences. This research design can reflect the essence of feeling, thinking, and ideas, which are conscious or unconscious in participants, through the analysis of intensive and extensive interviews [27]. This research design was adopted because it aims to explore how terminal cancer patients who have not clearly expressed a depressed mood or intense grief manage their feelings associated with anxiety and depression.

### Participants and setting

Japanese patients receiving palliative care at home or in PCUs were the participants. Purposive sampling was used to select the research participants. For recruiting patients who were aware of their terminal conditions, the clinics that operated according to the principle of hospice care were selected. Additionally, patients admitted to hospital PCUs after deciding to not take active treatments such as chemotherapy were selected. The study involved cancer patients who had (a) been advised that active treatment that aimed to provide a cure was judged ineffective, (b) been predicted to have a survival period of no more than six months, (c) no serious complications other than cancer, and (d) been judged by physicians or nurses as capable of engaging in interviews. Patients who exhibited a depressed mood or intense grief and were judged as unsuitable for participation by physicians or nurses were excluded from the study because of ethical considerations. The candidates for this research were selected by the nurse manager. The nurse manager explained to them the outline of the research and asked whether the researcher could visit to give a more detailed explanation. If consent was obtained, the researcher visited the candidate and explained the research.

Because it is important that people with experience provide numerous narratives in phenomenology, the sample size was intentionally reduced to include approximately one to six participants [28]. Sample size was set to five to six for each group in home care and PCUs. Among the 21 candidates who got internal consent, those who remarkably changed the medical condition were excluded; detailed research explanation was given to 19 candidates. Of these, three candidates thought that multiple interviews were difficult, and 16 participants submitted formal written consent form. Each participant was interviewed multiple times to ensure collection of detailed narratives. Given the physical and mental burden on participants, interview sessions were limited to approximately once a week. Four patients with whom at least two interviews could not be conducted because of exacerbation of their physical conditions were excluded from the analysis. One patient wished to discontinue the interview. Therefore, the analysis included 11 participants (six and five patients at home and in PCUs, respectively).

The interviews were unstructured. “How do you feel emotionally at the moment, and which views do you currently hold?” was included as the original question, inviting free narration. First, to build a relationship of trust and deepen the understanding of the participants, they were asked to share their life histories and cancer history. The contents concerning the current emotions spoken in this scene were also analysed. Then, the original question was presented. Deeper discussions were derived from the participants based on the preceding statement, compared to when asking questions, such as “Can you please tell me more about that?” or “How did you feel about …? ” If there was a story of the participant being shocked or suffering because of the terminal stage, it was interviewed carefully to identify how they think or feel now, and how they have reached the present idea.

In order to alleviate potential stress during the interview, family members were allowed to attend certain interviews on participants’ request. However, to encourage participants to discuss feelings they may not be comfortable disclosing to family members, family members were asked to not attend that particular interview. A total of three interviews were planned for each participant. The first interview was designed as a means to build a trusting relationship with the participant; the second interview focused on a deep disclosure of emotional reactions to the patient’s current health status; and the third interview focused on further disclosure in the absence of family members. However, no participant requested that a family member attend; thus, the minimum number of interviews conducted was two. To avoid significant differences between interviewers, all data were collected by a single researcher. The interviewer was an oncology nurse with a bachelor’s degree, trained to be sensitive to patients’ emotional reactions.

### Ethical considerations

The study was conducted with the approval of the Ethical Review Board for Medical Research Involving Human Participants at the Gunma University (approval number: 9–30) and National Hospital Organization Shibukawa Medical Center (approval number: 15–09-01). For the safety and peace of mind of both the interviewer and interviewees, a visiting nurse accompanied the interviewer during the first visit to help explain the nature of the study. The purpose of the study, voluntary nature of participation and the freedom to withdraw from the study at any time, and protection of personal information were explained verbally and in writing to participants, and their written consent for participation in the study was obtained. Consent was reconfirmed at the commencement of each interview. An interview was conducted only when a doctor, nurse, and nurse manager in charge judged that the interview was possible. Therefore, several interviews could not be conducted on the scheduled date. Each interview was generally planned for 60 min. Whether participants could continue with the interview was verbally verified every 15 min after the start of the interview. The interview time was extended only when the participant wished. Considering the participants’ physical and psychological burdens, nurses were asked to be present during interviews if participants so wished. There was no participant who required physicians and nurses extra support as the physical and psychological burden increased after the interview.

### Analysis

The interviews were recorded and transcribed verbatim, and the resultant data were analysed using the psychophenomenological approach [27], according to the following five-step procedure. In order to avoid inhibition of participants’ free speech and the researchers’ prejudice that “it will be done this way,” analysis was done after data collection was completed. The first step involved reading the transcripts several times to capture the sense of entire statements. The second step involved delimiting the transcripts via changes in thematic content, to identify meaning units. In the third step, each meaning unit was transformed from first- to third-person expression several times, using the phenomenological reduction and free imaginative variation. The unit was transformed from daily conversation into medical language, focusing on what patients had said or meant. The focus was on participants’ remarks concerning suffering and hope regarding life and death, clarifying how each participant felt, thought about, and recognized them. A transformation was applied from one to four times per meaning unit. It focused on disregarding researchers’ preconceived ideas about patients’ experiences. In this way, phenomenological reduction is indispensable for deriving meaning from the experiences of the interviewee rather than the researcher’s preconceptions [27]. In the fourth step, the transformed data was synthesized and the meaning units for each participant were structured, focusing on units that were essential to allow the phenomenon to manifest. Focusing on how a patient recognized the situation and how he/she faced life and death helped create an individual psychological structure for each participant. In the fifth step, all individual psychological structures were synthesized into a general psychological structure. During this analysis, attention was paid to characteristic words when facing death that were common to all participants. There is no saturation point with respect to deriving phenomenological meaning, to clarify the essence using phenomenological reduction and free imaginative variation [29].

### Trustworthiness

A 32-item checklist that “consolidated criteria for reporting qualitative research” was used [30]. At each stage, the researchers discussed the transformed meaning, individual psychological structures, and a general psychological structure, and the verbatim record was discussed until complete agreement was obtained. Additionally, the study was supervised by an oncology nursing expert and a psychological expert with thorough knowledge regarding qualitative research methods. In order to secure study credibility, persistent observations were conducted. Terminal participants were interviewed several times. The same researcher conducted all interviews in order to build a relationship of trust with each participant. Results were not reconfirmed with the participants as participants’ health problems and unexplored thoughts and emotions were subject to analysis. Peer debriefing was conducted in order to ensure credibility and dependability. Peer members were requested researchers familiar with qualitative nursing research qualified more than master’s degree. The materials included verbatim records, field notes, analysis notes, and notes on the researchers’ preconceptions. The preconceptions of researchers are bracketed in parentheses to ensure confirmability. The researcher’s prejudices were clarified by describing the researcher’s knowledge at the literature review stage of the present study. Due to transferability, purposive sampling was conducted in order to obtain a diverse sample with varying backgrounds, including age, illness period, palliative care period, etc. Finally, portions of the participants’ verbatim records were described below to provide concrete examples of extracted themes. All interviews were conducted in Japanese, and translation-back translation was conducted for purposes of the present manuscript.

## Results

### An outline of participants

Table 1 shows an outline of participants. The participants were three men and eight women aged between 53 and 94 years (mean = 72.6 years). The periods for which patients had been receiving palliative care at home and in PCUs ranged from 27 to 403 days (mean = 134.8 days) and from 14 to 107 days (mean = 35.8 days), respectively. When assessed using the palliative prognostic index, which is used to indicate prognosis [31], the survival period was predicted at up to three weeks for three participants.

**Table 1:** An outline of participants

	ID	Age	Sex	Type of Cancer	Illness Period (years)	Home Care/PCU Period (days)	Interview (times)	PS	PPI
HOME CARE	1	50s	F	Breast	7	27	3	0	0
	2	50s	F	Breast	11	126	3	4	2.5
	3	60s	F	Thyroid	5	403	2	4	6.5^a^
	4	80s	M	Lung	0.5	35	3	4	8.5^a^
	5	80s	F	Ovarian	13	158	3	2	0
	6	90s	M	Prostate	17	60	5	3	3.5
PCU	7	50s	M	Appendix	0.3	28	3	4	4.5
	8	50s	F	Breast	12	16	3	1	0
	9	70s	F	Lung	0.4	107	2	4	6.0^a^
	10	80s	F	Breast	21	14	3	3	4.5
	11	80s	F	Pancreas	0.3	14	3	3	4.5

Note: Illness Period at the first interview

Home Care / PCU Period at the first interview

PS: Performance status at the end of interviews

PPI: Palliative Prognostic Index at the end of interviews

^a^PPI score greater than 6.0 indicates 3 week survival

Each participant was interviewed between two and five times. The participant who interviewed five times continued the interview because he strongly hoped that it would take time to talk about life history and would like to speak more. Each interview session lasted between 22 and 135 min (mean = 61.4 min). The overall duration of interview time for individual participants ranged between 90 and 517 min (mean = 184.3 min) (33 interviews and overall interview time of 2027 min for all participants).

### The expressions of “I have to accept …”

The following five themes emerged for the experience of managing feelings associated with anxiety and depression when facing death in patients with terminal cancer at home or in a PCU: “I have to accept that I have developed cancer,” “I have to accept the undeniable approach of my own death,” “I have to accept my need for assistance,” “I have to accept this unsatisfactory circumstance” and “I have to accept this as my destiny and an outcome of my life.”

When describing how they had managed their feelings when facing death, participants tended to use the Japanese word “Shouganai” numerous times to express “I have to accept” or “it cannot be helped,” thereby attaching different meanings to the term. The words of the participants are shown in *italics*.

### I have to accept that I have developed cancer

The participants were distressed and wondered why they had developed cancer, which reduced their independence and threatened their lives, and used the phrase “I have to accept.” Participants attempted to control the frustration and despondence resulting from the development of cancer by telling themselves “I have to accept” it or “There is nothing that can be done.”

One woman looked back on the development of cancer, which had caused leg paralysis, and her married life and became stressed, felt sorry for herself and wept, and wondered why it had happened to her. However, she felt that weeping would not change her situation, but she had been saved by medical professionals and care staff, whom she was able to meet because she was ill, and she tried to accept her situation.

*Why did only I catch such a sickness? There is nothing that can be done [pause]. I got married to a struggling husband and got sick. I have no luck [bitter smile][pause]. I sometimes think so [pause], but I think that I am blessed, because nurses and home care workers are nice people. (Participant 3, interview 1).*

*I thought, ‘why did I become so sick?’ [pause], but nothing can be helped. I will be possessed by this illness for the rest of my life [crying] [pause]. There is no choice but to cry every day [pause]. Even if I cry, my illness does not go away. I have convinced myself. (Participant 3, interview 2).*

### I have to accept the undeniable approach of my own death

The participants tended to think that it was “unavoidable” that their tumors would grow when no active treatment, such as chemotherapy, was used. The participants felt that their symptoms were worsening and their physical power was deteriorating. The participants were afraid of death and the process of death and earnestly desired to live but felt that death was imminent. To escape their distress, they attempted to accept the situation by thinking that all lives are finite and death had to be accepted.

One woman felt that it was inevitable that her cancer would advance when she was no longer receiving chemotherapy, but she was also afraid of a possible relapse and the intense suffering that she had experienced during chemotherapy.

*Three months after my anti-cancer drug therapy was discontinued, I underwent an X -ray. At that time, my tumor had grown by one level. I thought that this was inevitable and that there was nothing that I could do. I am now afraid that the continuing and very severe suffering that I experienced during the anti-cancer therapy period may recur at any time. (Participant 9, interview 1).*

One woman’s cancer had recurred, and she felt her physical strength reducing every day. Considering her age, she felt that her impending death could not be helped, but she had not given up. When her cancer returned, she initially gave up and accepted death by telling herself this, but after some time had passed, she felt that she might be able to live longer and was aware that she could not give up living, because she feared death.

*I think that it cannot be helped, because I will not be able to escape from death coming soon; however, it is not easy to wait for that day to come [pause] … but I have already reached such an [old] age. The true story is, I do not think I could die anymore. If I said that I was not afraid to die, it was a lie, because I believed more strongly that I would not recover at that time [of recurrence] than I do now. I seriously thought that I could die [pause]. It could not be helped. However, time does not go quickly [pause]. Chaos comes to my heart [pause]. Human beings are selfish [short laugh]. (Participant 5, interview 2).*

### I have to accept my need for assistance

Considering primarily the burden on their families, the participants asked themselves about the value of their lives, wondering whether it was worthwhile to continue to live while troubling their families.

One woman was paralyzed from the waist down after unsuccessful treatment for recurrence. She accepted that she could not move and had to live with nursing care, by telling herself she had to accept her situation. She recognized that although she was a burden to her husband, the fact that she continued to live kept her parents from feeling unhappy.

*During the first month after I was discharged from the hospital, my husband gradually became irritated while playing previously unexperienced roles, such as exchanging my diapers and helping me to eat and brush my teeth. Lying on my bed, I felt sorry for my husband each time he cared for me. I had no idea how long such a life would continue, but within about a month of the start of that life, both my husband and I gradually became familiar with care. Now I feel that I have to accept this way of living. Emotionally, I find it inevitable. Even if I become depressed, no one will help me. So, I have to accept it (skipped). I cannot die now if I imagine how deeply my parents will feel sad. (Participant 2, interview 3).*

### I have to accept this unsatisfactory circumstance

The patients in PCUs felt dissatisfied with having to stay in the hospital despite wishing to live at home, or family members failing to prioritize their feelings or desires. Patients living at home rarely reported this discontent.

One woman wished to live at home but was aware of the need for hospital admittance, because her family members were unable to care for her. During her hospital stay, her family members did not visit her ward as frequently as she would have liked. She felt lonely and dissatisfied but attempted to suppress these feelings, telling herself that it was inevitable, because her family members were busy.

*At home, family members cannot take care of me. So, I thought that it was inevitable for me to become hospitalized. My son was busy and unable to visit me last weekend. I have to wait another week before seeing him. I imagine he is busy as a school teacher. As he is not a classroom teacher, he may not be so busy, but then he may be busy with other work related to school. So, because he is busy, I have to accept that he cannot visit me frequently. It is boring to remain in bed. I am waiting for a family member to visit my ward. (Participant 11, interview 1).*

### I have to accept this as my destiny and an outcome of my life

The participants were aware that they could not do anything themselves to fight their disease or the undeniable approach of death and needed support, and those around them did not behave as they would have liked.

One woman had a religious background and was able to think “It cannot be helped,” because she could not go against fate. She recognized that she had had relatively low anxiety and distress levels after developing cancer. She thought that her life would end soon because she had cancer. However, from her experience of recovering after facing death once, she considered her life as “given life” and wished to do what she could for others.

*As I had no control over my fate, I could not be helped, even if I was writhing and struggling [pause]. Both my husband and I believe in Buddhism. I was released so much from anxiety and so on. My heart was in very good condition. … When I became ill, I had a feeling that it was for a lifetime, but at that time I did not die. I got a life again like this. So in my next life, I want to do something for people. (Participant 1, interview 3).*

One man described the situation in which he gradually became unable to care for himself as being half dead. While he wished to die soon to escape this situation, he told himself that it could not be helped, because life and death are part of nature, and he could not go against nature.

*This situation is like a half-killing snake. If this is the case, I want to pass away soon. There is no reversal from here. It cannot be helped, because people cannot go against nature. My children are coming sometimes now, but if my life drags on, they will feel stressed. I think that the end of life is like this. There are not many books that teach us about such feelings at the end of life. The end of life’s mental feeling I have now is that there is a waterfall [to die], and I have to fall there. I cannot escape. This is a living hell. People with the same circumstances are all feeling the same. (Participant 6, interview 5).*

Some participants believed that their current situation was “inevitable” as an outcome of their past deeds. One woman experienced exacerbation of her symptoms because of cancer progression and was told by the physician that she might die. While she was going through this experience, problems that had been masked previously in relationships with family members began to surface. She reviewed the course of her life with her husband (now receiving icy stares from her children) and her own situation, full of concern, and reached the conclusion that this was the outcome of her past deeds.

*I feel that the ways in which we deal with our children become evident in this situation. It is too late to cry for past mistakes. That was what we did in the past. Although I feel sympathy for my husband, it is difficult to deny that he truly did nothing for the sake of the children. So, I think that the current situation has to be accepted. (Participant 8, interview 3).*

## Discussion

Terminal cancer patients at home or in PCUs experience agony over “why they developed cancer,” a major source of their current crisis, and feelings associated with an awareness of disease progression and approaching death. While participants were dissatisfied with needing care and unpleasant reactions from family members, they attempted to accept their circumstances, thereby managing feelings associated with anxiety and depression.

This study demonstrated that patients told themselves that “I have to accept it” to manage their feelings when facing death. Thus, they felt thankful for being alive and around people, and they were finding positive meaning even in such a serious situation. The Japanese expression, “Shouganai,” which is equivalent to “I have to accept it” or “it cannot be helped” in English, is used in situations in which there is no means of resolving an issue [32]. This term is used on a daily basis in Japan. The idea of “it cannot be helped” has been examined in various fields ranging from the extraordinary huge-scale disasters such as earthquakes [33] and nuclear incident [34], economics [35], education [36], and individual identity [37]. Japanese have a fatalistic idea “Shouganai” towards they cannot change anything.

It is not only Japanese patients who tell themselves “I have to accept it” in a difficult situation that cannot be controlled. This is one of the cognitive reinterpretation and adaptive coping skills, which involves “reinterpreting adverse experiences to find meaning and benefit” [38]. Thereby, the patient maintains self-efficacy for coping with cancer [39]. The results of this research have universality and thus are applicable to patients with terminal cancer globally regardless of culture or race.

### Facing cancer and their own deaths, which they had been trying to avoid

The participants lived in a state of distress, wondering why they had developed cancer, which reduced their independence and threatened their lives, and told themselves that they had to accept that they had developed the disease.

This is consistent with spiritual suffering resulting from a sense of unfairness and wondering, “Why was it me who developed such a disease?” [40]. Cancer is a major cause of death in many countries including Japan [41]. Despite statistics, many people believe that life declines very, very gradually and they will eventually die, but they feel that today and tomorrow are no different from yesterday. Therefore, although they comprehend the notion that that anyone could develop cancer, they cannot understand why they did so.

Patients looked back on their lives in distress, believing that events that caused stress or an unsuccessful struggle with cancer could have caused their current circumstances. However, they felt that the development of cancer and life events that did not turn out as they expected were “I have to accept it” and therefore accepted their past lives.

The participants were afraid of death and the process of death and earnestly desired to live, but they were aware of their impending death and felt distressed. To escape this distress, they attempted to accept the situation by thinking that all lives are finite.

The feeling of not wanting to die is instinctive and felt by everyone, but many patients “give up” when their bodies become weak following disease progression. Their feelings swing between acceptance of death (i.e., “I have to accept it”) and the desire to live, and this is part of the preparation for death.

Young patients and those with a short disease duration are expected to feel higher levels of distress, because they consider age and disease duration criteria for accepting death. Following a diagnosis of cancer, most patients realize that death is real and become resigned to it during the process of treatment. However, they consider having lived after being resigned to death as “life prolonged by someone.” They then endeavor to prepare for imminent death because it is “it cannot be helped.”

### The unsatisfactory body and circumstances

The participants were in distress and wondered whether it was worthwhile continuing to live while burdening their families but tried to accept themselves with reduced independence and a need for nursing care by saying “it cannot be helped.”

Patients with terminal cancer are aware that they are steadily becoming weaker by the day and experience the inability to do what they could once do as a matter of course. In addition, some patients suddenly require long-term care because of paralysis caused by spinal metastasis. Moreover, the number of activities that they are unable to perform increases steadily, and they experience loss and distress repeatedly. Close attention has recently been paid to cognitive behavioral therapy [42] and Mean-Centered Psychotherapy-Palliative Care [43] to help patients deal with spiritual suffering (e.g., despair and the loss of meaning in their lives). By accepting themselves with reduced independence, patients notice that “It is important to maintain self-determination ability if they cannot engage in self-care.”

In addition, a good death in Japan is characterized by an emphasis on interpersonal relationships with family members and loved ones [44]. A similar trend has been observed in Korea (a country in a cultural zone that is close to that of Japan) [45], and others [46, 47], where terminal cancer patients rate “not being a burden to the family” as very important. By believing that “I have to accept it” to receive care from others, patients reconsider themselves from the perspectives of others. Thus, they realize that there are still things they can do for their family and the others and find meaning in life.

Patients in PCUs were dissatisfied with hospitalization despite their desire to live at home with family members, who failed to prioritize to their feelings and desires. However, they attempted to suppress this discontent by telling themselves that this should be accepted, because family members had their own issues to deal with.

Many patients cannot live out their final days at home, even if they so desire. According to the Vital Health and Social Statistics Office [48], 76.6% of people die in hospital or a medical clinic, while only 12.7% die at home, in Japan. According to a review of English papers conducted in 2014, between 40 and 85% of patients with terminal cancer wished to die at home, but the rate of home death ranged from only 20 to 66% [49]. Moreover, the results of a previous survey indicated that 43.7% (*n* = 191) of patients wished to die at home, but only 13.6% (*n* = 26) did so in Korea [50].

Patients with terminal cancer generally feel lonely in hospital, and this feeling is stronger in those without visitors and/or family support [51]. It is therefore necessary for nurses to listen to the complaints of patients who feel lonely, remaining close to them.

### Consciousness of the transcendental existence of destiny

The patients attempted to accept their disease, the undeniable approach of death, their need to receive assistance, and the fact that those around them did not behave as they would have desired as “uncontrollable destiny” reflecting the outcomes of their past deeds. They attempted to follow the natural course through acceptance of their circumstances.

Patients with terminal cancer endeavor to calm the troubled waters of the mind and accept death by yielding to an extraordinary higher power (i.e., “life given by someone”). For this reason, they are deeply grateful to the people whom they have met, and feel that they have not only distress but also happiness in their lives.

Some participants stated that the “higher power” was Buddha, but for many, it was an inconclusive, vague concept that included “nature.” Numerous people from around the world including the Japanese have scientism-based worldviews. The rate of atheist, agnostic, and nonbeliever exceeds 80% in some countries, with the highest in the order of Sweden, Vietnam, Denmark, Norway, and Japan [52]. This perspective does not appear to be specific to Japanese people and is observed frequently in patients with terminal cancer from other cultural and racial backgrounds.

Patients with terminal cancer who receive palliative care at home or in PCUs attempted to suppress their repulsion toward the development and progression of cancer and the decline in their autonomy, those around then failing to behave as they would have desired, their own approaching death, and the destiny that induced such problems, by telling themselves that they had to accept it. In this way, these patients attempted to achieve mental well-being under difficult and severe circumstances. Thus, patients reinterpret the circumstances and life experiences. They experience gratitude towards their life, self-determined, and families, and can thereby find meaning in their life.

## Conclusion

The purpose of this study was to explore how patients with a terminal illness manage feelings associated with anxiety and depression. To this end, the narratives of 11 patients were analyzed via qualitative methods, and the following five themes were extracted: “I have to accept that I have developed cancer,” “I have to accept the undeniable approach of my own death,” “I have to accept my need for assistance,” “I have to accept this unsatisfactory circumstance” and “I have to accept this as my destiny and an outcome of my life.”

When patients realize that their death is approaching, they look back on their lives, and their struggle with cancer, and explore reasons for their current suffering. The new finding of this research is that terminal patients try to accept this “uncontrollable destiny” by accepting a limited life, an unsatisfactory body, and distressing circumstances as part of “I have to accept it.” Patients are reconciled with their lives and trying to stabilize their mind each day. “I have to accept it” will be expression in a sense that it tells themselves more than to communicate to others.

Nurses need to approve patient efforts to manage feelings associated with anxiety and depression. This could be accomplished by nurses better comprehending patients’ complicated mental patterns expressed in their daily languages. Finally, the present study suggests the need for nurses to capture the meaning of “I have to accept it” for patients in order to alleviate distress at the end of life.

The study included patients who received care either at home or in PCUs. Therefore, further studies involving patients in general hospitals and other establishments are required.

## Abbreviation

PCUs: Palliative care units
